# Supplementary material for: A Novel Glass Fiber Coated with Sol–Gel Poly-Diphenylsiloxane Sorbent for the On-Line Determination of Toxic Metals Using Flow Injection Column Preconcentration Platform Coupled with Flame Atomic Absorption Spectrometry
Source: Molecules. 2020 Dec 22;26(1):9. doi: 10.3390/molecules26010009 (PMC7792807; doi:10.3390/molecules26010009)
Supplement: Supplementary file 1 [file molecules-26-00009-s001.pdf]

molecules

MDPI

Supplementary Material

Caption of Figures:

Figure S1. Preparation of the microcolumn packed with sol-gel PDPS coated glass fiber: a) cutting of glass fibers; b) packing with GF coated with sol-gel PDPS.
Figure S2. Installation of the GFSE microcolumn in the FIAS-400 system.
Figure S3. Effect of sample flow rate on the absorbance of 100.0 μg·L^{-1} Pb(II) and 100.0 μg·L^{-1} Cr(VI). All other experimental parameters as in Table 1.
Figure S4. Effect of elution flow rate on the absorbance of 100.0 μg·L^{-1} Pb(II) and 100.0 μg·L^{-1} Cr(VI). All other experimental parameters as in Table 1.
Figure S5. Effect of preconcentration time on the absorbance of 100.0 μg·L^{-1} Pb(II) and 100.0 μg·L^{-1} Cr(VI). All other experimental parameters as in Table 1.

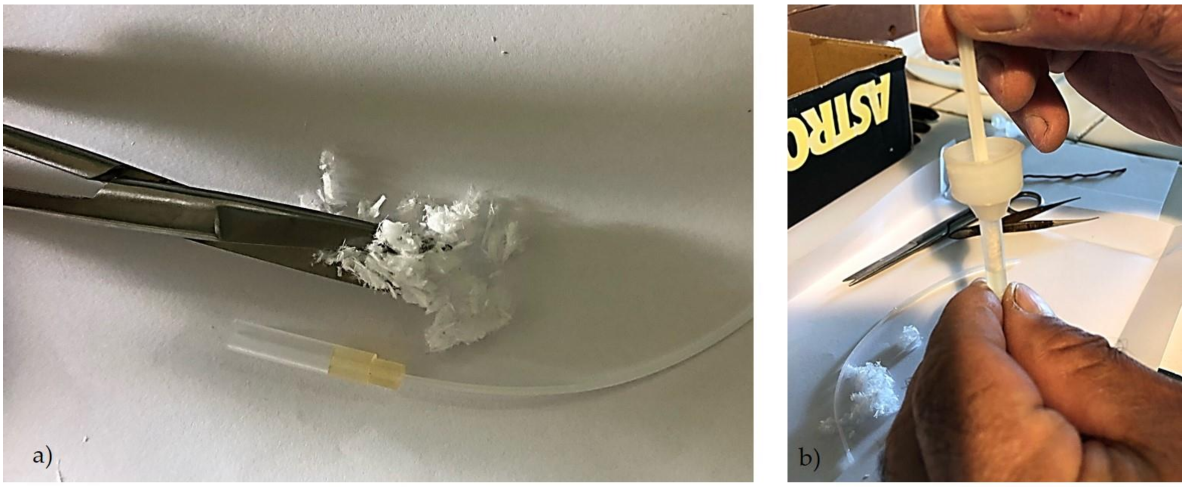

Figure S1. Preparation of the microcolumn packed with sol-gel PDPS coated glass fiber: a) cutting of glass fibers; b) packing with GF coated with sol-gel PDPS.

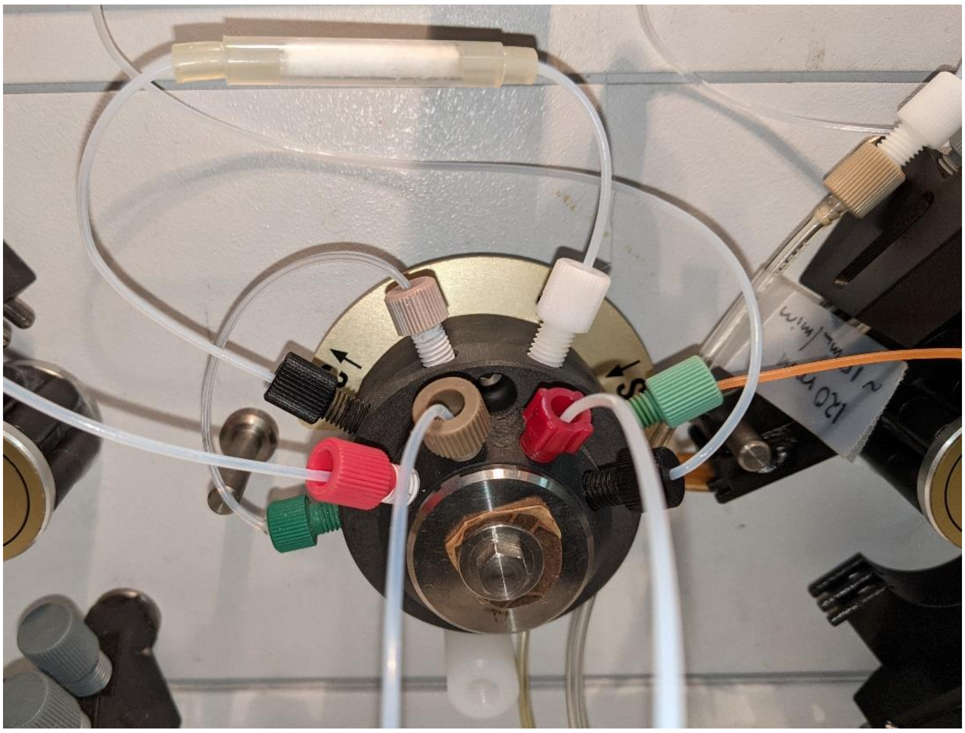

Figure S2. Installation of the GFSE microcolumn in the FIAS-400 system.

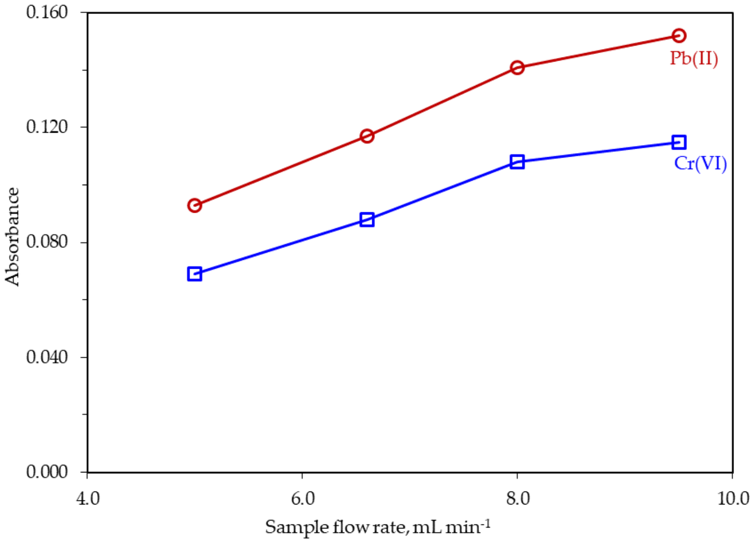

Figure S3. Effect of sample flow rate on the absorbance of 100.0 μg·L^{-1} Pb(II) and 100.0 μg·L^{-1} Cr(VI). All other experimental parameters as in Table 1.

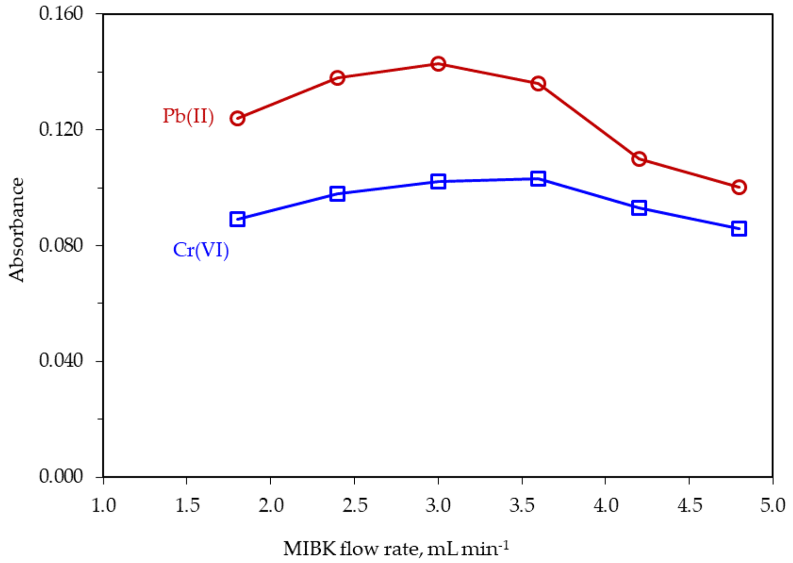

Figure S4. Effect of elution flow rate on the absorbance of 100.0 μg·L^{-1} Pb(II) and 100.0 μg·L^{-1} Cr(VI). All other experimental parameters as in Table 1.

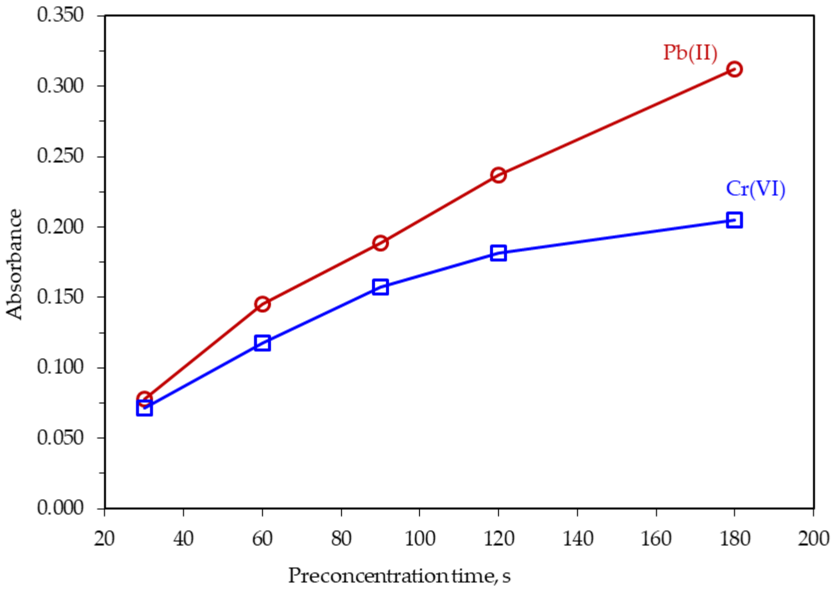

Figure S5. Effect of preconcentration time on the absorbance of 100.0 μg·L^{-1} Pb(II) and 100.0 μg·L^{-1} Cr(VI). All other experimental parameters as in Table 1.
